# An Effective Modification Strategy to Build Multifunctional Peptides Based on a Trypsin Inhibitory Peptide of the Kunitz Family

**DOI:** 10.3390/pharmaceutics16050597

**Published:** 2024-04-27

**Authors:** Ying Wang, Daning Shi, Wanchen Zou, Yangyang Jiang, Tao Wang, Xiaoling Chen, Chengbang Ma, Wei Li, Tianbao Chen, James F. Burrows, Lei Wang, Mei Zhou

**Affiliations:** 1 Natural Drug Discovery Group, School of Pharmacy, Queen’s University Belfast, Belfast BT9 7BL, Northern Ireland, UK; ywang155@qub.ac.uk (Y.W.); wzou02@qub.ac.uk (W.Z.); yangyang.jiang@qub.ac.uk (Y.J.); t.wang@qub.ac.uk (T.W.); c.ma@qub.ac.uk (C.M.); t.chen@qub.ac.uk (T.C.); j.burrows@qub.ac.uk (J.F.B.); l.wang@qub.ac.uk (L.W.); m.zhou@qub.ac.uk (M.Z.); 2 Chinese Academy of Agricultural Sciences, No.12 Zhongguancun South Street, Haidian District, Beijing 100081, China; shidaning@caas.cn; 3 College of Chinese Medicinal Materials, Jilin Provincial International Joint Research Center for the Development and Utilization of Authentic Medicinal Materials, Jilin Agricultural University, Changchun 130118, China

**Keywords:** Kunitz-type peptides, multifunctional peptides, secondary structure, antimicrobial activity in vitro and in vivo, antiproliferative activity, apoptosis

## Abstract

Peptides with antimicrobial activity or protease inhibitory activity are potential candidates to supplement traditional antibiotics or cancer chemotherapies. However, the potential of many peptides are limited by drawbacks such as cytotoxicity or susceptibility to hydrolysis. Therefore, strategies to modify the structure of promising peptides may represent an effective approach for developing more promising clinical candidates. In this study, the mature peptide OSTI−1949, a Kunitz-type inhibitor from *Odorrana schmackeri*, and four designed analogues were successfully synthesised. In contrast to the parent peptide, the analogues showed impressive multi-functionality including antimicrobial, anticancer, and trypsin inhibitory activities. In terms of safety, there were no obvious changes observed in the haemolytic activity at the highest tested concentration, and the analogue OSTI−2461 showed an increase in activity against cancer cell lines without cytotoxicity to normal cells (HaCaT). In summary, through structural modification of a natural Kunitz-type peptide, the biological activity of analogues was improved whilst retaining low cytotoxicity. The strategy of helicity enhancement by forming an artificial α-helix and ß-sheet structure provides a promising way to develop original bioactive peptides for clinical therapeutics.

## 1. Introduction

Amphibian skin secretions are an abundant source of bioactive compounds as they are rich in bioactive constituents, such as alkaloids, biogenic amines, proteins, and peptides. Notably, peptides have emerged as the predominant molecular class within the secretions of frog skin, particularly peptides with antimicrobial activity or protease inhibitory activity, which are potential candidates as alternatives to traditional antibiotics or cancer chemotherapies [1]. Proteases, known to be present in all organisms, primarily facilitate protein turnover in living systems through their biocatalytic function in protein metabolism. Given the pivotal role of proteins in sustaining homeostasis, proteases are essential in regulating physiological activities. The dysfunction of proteases has been linked to various pathological conditions in humans and animals. Consequently, protease inhibitor peptides, known for their ability to obstruct protease–substrate interactions, have promising prospects in drug discovery [2]. Numerous protease inhibitor peptides with a molecular weight under 10 kDa have been discovered in plant sources and the skin of amphibians. These peptides display an array of bioactivities including inhibitory, antiviral, antifungal, antibacterial, anticancer, anti-inflammatory, antinutritional, and immunomodulatory activities. Certain protease inhibitor peptides exhibit dual functions, encompassing both protease inhibition and antimicrobial activity [3,4,5,6]. 

The first discovery of a Kunitz family member was the soybean Kunitz trypsin inhibitor (SKTI) [7], and similar sequences were subsequently identified in various plants. Meanwhile, certain members of the Kunitz family were also found in frog skin secretions such as Kunitzin-RE and Kunitzin−OS [8], OGTI [9], and Kunitzin−OV [10]. According to the MEROPS database [11], Kunitz-type trypsin inhibitors (KTIs) are categorised into two groups depending on their origin in either animals or plants. These inhibitors exhibit a specific loop of “-RCKAAFC-”, which is a completely conserved region of seven amino acids, with the Arg residue, which acts as the principal trypsin cleavage site, adjacent to the disulfide loop. When the Lys residue is substituted with Phe in the P1 position, the inhibitory activity of the peptides against trypsin substantially decreased. Additionally, the Cys-Cys domain without the Arg residue had greatly decreased anti-trypsin activity [8,10]. 

It is hypothesised that these inhibitors may serve multiple roles such as storage proteins, regulators of endogenous proteases, and contributors to defence mechanisms against pests and pathogens [12]. These functions include anti-pest properties achieved by inhibiting protease activities within insects, thereby impeding the digestion of proteins [13], and they can potentially contribute to cancer prevention by inhibiting the signalling cascades of mitogen-activated protein kinase (MAPK)-dependent urokinase [14]. Additionally, through the inhibited MAPK-dependent signalling pathways, these peptides play a role in inhibiting the expression of anti-inflammatory mediators [15]. The identified KTIs also showed strong anticancer activity in an animal model by effectively obstructing activators of urokinase-type plasminogen, thereby suppressing the invasion of ovarian cancer cells [16,17]. 

In this study, the peptide OSTI−1949, a KTI, was reported initially as an antimicrobial protein precursor named ranatuerin-2SHb with 70 amino acids (GenBank: ADE48807). An alignment analysis identified the mature peptide, and the biological activities of OSTI−1949 were assessed. Several analogues were subsequently designed, and their enhanced biological activities such as antimicrobial activity and antiproliferative activity against cancer cell lines were observed. These multifunctional peptides have potential as clinical therapeutics, and they show that the structural modification strategies used are effective and useful ways to improve natural protease inhibitors.

## 2. Materials and Methods

### 2.1. Peptide Design and Solid-Phase Peptide Synthesis (SPPS)

Based on previous research [18,19,20], the analogues were modified strategically by enhancing the helicity of secondary structures or forming an artificial α-helix and ß-sheet at the N-terminus of protease inhibitor peptides (Table 1). After modification, an automated solid-phase peptide synthesiser (Protein Technologies, Tucson, AZ, USA) was used to synthesise the parent peptide and analogues. Every amino acid of the peptide was weighed, and 0.3 mmol × 2.5, 2-(1H-benzotriazole-1-yl)-1,1,3,3-tetramethyluronium hexafluorophosphate (HBTU, a coupling reagent) was also weighed and mixed with the amino acids in each vial. The synthetic peptides were then cleaved from the resin in a mixture solution containing 94% trifluoroacetic acid (TFA), 2% ddH_2_O, 2% thioanisole (TIS), and 2% 1, 2-ethanedithiol (EDT). Finally, the filtered solution was reverse extracted using ether and centrifuged, after which, the ether was drained and the product was dried in a ventilated area. The resulting lyophilised peptides were stored at −20 °C.

### 2.2. Prediction of Physicochemical Properties and Secondary Structure

The peptides’ physicochemical properties were computed using the online proteomic bioinformatic resource BACHEM (https://www.bachem.com/knowledge-center/peptide-calculator/ (accessed on 15 February 2023)). The secondary structure prediction of the peptides was performed by using the online web server PEP-FOLD 3.5 (https://mobyle.rpbs.univ-paris-diderot.fr/cgi-bin/portal.py#forms::PEP-FOLD3 (accessed on 13 February 2023)).

### 2.3. Purification and Identification of Peptides

Reverse-phase high-performance liquid chromatography (RP-HPLC) with an aeris widepore xb-c18 column (250 × 10 mm, Phenomenex, UK) was used to purify the crude synthetic peptides according to their distinctive polarity or hydrophobicity. The mobile phase consisted of two solutions of ddH_2_O and 0.5% TFA as Solution A and 80% acetonitrile, 19.5% ddH_2_O, and 0.5% TFA as Solution B. 

Matrix-assisted laser desorption ionisation time-of-flight mass spectrometry (MALDI-TOF MS) (Voyager DE, Perseptive Biosystems, Framintham, MA, USA) was used to analyse and identify the synthetic peptides. The matrix solution was a solvent of CHCA (10 mg/mL), which consisted of 70% acetonitrile, 30% water, and 0.1% TFA. Two microlitres of the HPLC fractions and 1 μL of the CHCA solution were loaded onto the plate, and the plate was uploaded after the drops dried. The mass/charge ratios (*m*/*z*) were recorded to identify the target peptide.

### 2.4. Peptide Secondary Structural Analysis

The secondary structures were analysed using a JASCO J815 circular dichroism (CD) spectrometer (Jasco, Essex, UK). Peptides were dissolved to a concentration of 100 μM with an NH_4_Ac solution (20 mM) acting as the stock solution. Then, the stock solution was mixed with an equal volume of ddH_2_O and TFE, respectively, to create working solutions, which were used to mimic an aqueous environment and membrane environment. The peptide working solutions were scanned at wavelengths ranging from 190 to 260 nm in a quartz cuvette with a thickness of 1 mm. The scanning speed was set at 200 nm/min, and the bandwidth and data pitch were 1 nm and 0.5 nm, respectively.

### 2.5. Trypsin Inhibition Determinations

Peptide was dissolved to make a stock solution at 1 × 10^4^ μM with phosphate-buffered saline (PBS), and then diluted to a series of working concentrations ranging from 1 to 1000 μM. To each well of a black 96-well plate, a working peptide concentration, 180 μL of substrate (50 μM in PBS), and 10 μL of trypsin (0.1 μM in 1 mM HCl) were added up to a final volume of 210 μL in a dark environment. The trypsin was added last, and the plate was measured immediately. The fluorescence intensity of each well was recorded for 30 min at every 30 s using a FLUOstar OPTIMA plate reader (BMG Labtech, Ortenberg, Germany). As the substrate of trypsin in this assay was Phe-Pro-Arg-AMC (Bachem, Merseyside, UK), the emission and excitation wavelengths were 460 nm and 395 nm, respectively, and were measured at 37 °C. The curves of the trypsin inhibitory activity were generated using the Morrison equation in Prism 9 (the working concentration of the substrate, [S] = 42.86 μM; the working concentration of trypsin, Et = 0.0020 μM; trypsin kinetic constant, Km = 41.07 μM). 

### 2.6. In Vitro Antimicrobial Assays

The values of MIC and MBC were used to determine the antimicrobial activity of the peptides. The minimum inhibitory concentration (MIC) represents the lowest concentration at which the peptides can inhibit visible bacterial growth. The minimum bactericidal concentration (MBC) indicates the lowest concentration at which the peptides can kill all bacteria. Ten bacteria strains were selected for this assay including *Escherichia coli* (*E. coli*, ATCC CRM 8739) (*E. coli*, BAA 2340) (*E. coli*, NCTC 13846), *Pseudomonas aeruginosa* (*P. aeruginosa*, ATCC CRM 9027), *Staphylococcus aureus* (*S. aureus*, ATCC CRM 6538), *Candida albicans* (*C. albicans*, ATCC 10231), *Enterococcus faecium* (*E. faecium*, NCTC 12697), methicillin-resistant *Staphylococcus aureus* (MRSA, NCTC 12493), *Klebsiella pneumonia* (*K. pneumonia*, ATCC CRM 43861), and *Acinetobacter baumannii* (*A. baumannii*, BAA 747). 

The bacteria were taken from a −20 °C freezer and incubated in a flask with culture medium on an orbital shaker at 120 rpm/min at 37 °C (or 26 °C for yeast) for 16–20 h. Subsequently, 0.5 mL of the cultured bacteria was transferred into McCartney bottles containing 20 mL of broth medium for sub-culturing under the same conditions. The sub-cultured bacteria were diluted 200 times with fresh medium to achieve a working bacterial density of 5 × 10^5^ CFU/mL based on the optical density (OD) at a wavelength of 550 nm. The peptides were dissolved in dimethyl sulfoxide (DMSO) at concentrations ranging from 100 μM to 51,200 μM and tested against the diluted bacteria, along with positive controls of norfloxacin or amphotericin B and negative and vehicle controls. After loading different media into a 96-well plate, the plate was incubated for 20–24 h, and OD values were measured at 550 nm using a plate reader. The minimal inhibitory concentration (MIC) and minimum bactericidal concentration (MBC) were determined as the lowest peptide concentrations inhibiting visible bacterial growth and resulting in no bacterial growth, respectively.

### 2.7. Time-Killing Kinetic Assays 

The time-killing kinetic assay was executed to explore the killing efficiency of the peptides against bacteria. The concentrations of the peptides tested were at values of 1 × MIC, 2 × MIC, and 4 × MIC; the three strains tested in this assay were *Escherichia coli* (ATCC CRM 8739) and two drug-resistant strains of *Escherichia coli* (NCTC 13846 and BAA 2340). The bacteria were cultured using the same method as that employed in the antimicrobial activity determination assay. The bacteria–peptide mixture was diluted 10, 100, and 1000 times, and then 10 μL of the mixture at four different bacterial densities were inoculated onto plates with solid culture medium at the time points of 0, 5, 10, 15, 30, 60, 90, 120, and 180 min. All colonies were counted and recorded after the seeded plates had been incubated at 37 °C for 24 h. 

### 2.8. SYTOX Green Permeability Assays

The SYTOX^TM^ Green Nucleic Acid Stain (ThermoFisher Scientific, Waltham, MA, USA) assay was used to investigate the antibacterial mechanism of the peptides as they cannot cross intact membranes, but can easily penetrate the damaged membranes characteristic of dead cells.

At first, the bacteria (*E. coli*, NCTC 13846/BAA 2340) were cultured with TSB in an orbital shaker at 37 °C overnight. After being sub-cultured for 2 h, the bacteria were centrifuged at 1000× *g* for 10 min at 4 °C; then, the culture medium was discarded. The bacteria at the bottom of the tube were washed gently twice with 5% TSB (in 0.85% NaCl). After a third wash, the bacteria were diluted with the same solution until they reached the logarithmic growth phase in which the OD value just reached 0.70 at a wavelength of 590 nm. Subsequently, 40 μL of the peptide solutions at the concentrations of MIC, 2 × MIC, and 4 × MIC and 50 μL of the bacterial suspension were loaded into a black 96-well plate and then incubated for 2 h at 37 °C. Finally, 10 μL of SYTOX^TM^ green nucleic acid stain (5 μM) was added to the plate and the mixture was incubated at 37 °C under dark conditions for another 5 min. The intensity of fluorescence was detected by Synergy HT (BioTech, Minneapolis, MN, USA) at wavelengths of excitation and emission of 485 and 528 nm, respectively. Bacterial medium with 5% TSB acted as the negative control; bacterial medium treated with a melittin peptide solution (8 μM) was set as the positive control; 5% TSB alone was the blank control.

### 2.9. In Vivo Antimicrobial Activity Determination 

The larvae of *Galleria mellonella* were used to evaluate the in vivo antibacterial activity of the peptides [21,22]. In this assay, the larvae (250 ± 25 mg) (Livefood UK Ltd., Rooks Bridge, UK) of the infected group were infected with 10 μL of a bacterial suspension (1 × 10^7^ CFU/mL). After one hour, 10 μL of a peptide solution was injected into each infected larva. As a positive control, infected larvae received 20 mg/kg of rifampicin. The vehicle control consisted of uninfected larvae administered 10 μL of PBS. All larvae were then monitored every 24 h for a duration of five days.

### 2.10. Haemolysis Assays

The purpose of the haemolysis assay is to evaluate the cytotoxicity of peptides against horse red blood cells in vitro. Erythrocytes were obtained from defibrinated horse blood and then washed with PBS to produce a 4% suspension in PBS as a working solution. The peptide was dissolved in DMSO to prepare a stock solution; then, it was diluted with PBS to working concentrations ranging from 32 μM to1024 μM (DMSO was less than 1% in the total volume). Then, 100 μL of peptide solutions at different concentrations and 100 μL of the 4% erythrocyte suspension were mixed in tubes and the tubes were placed in an incubator at 37 °C for 2 h. Afterwards, these tubes were centrifuged for 10 min at 930× *g* and the supernatant in each tube was carefully transferred into a 96-well plate. The lysis of red blood cells in the plate was read at 470 nm using a Synergy HT plate reader (BioTech, Minneapolis, MN, USA). The positive control was the 4% erythrocyte suspension treated with 1% Triton X-100 (Sigma-Aldrich, St. Louis, MO, USA), and the negative control contained the 4% erythrocyte suspension and a 1% DMSO solution (diluted with PBS). 

### 2.11. Antiproliferation Assays

This assay was performed to study the antiproliferative activity of the peptides against human cell lines by using the characteristics of MTT, which, in live cells, can be converted into a formazan that does not dissolve in water. The tested human cell lines in this assay included cancerous and non-cancerous cell lines: a human breast cancer cell line (MCF-7), a human lung carcinoma cell line (H838), a human colorectal carcinoma cell line (HCT116), and a human glioblastoma astrocytoma cell line (U251MG); these cell lines were incubated with the peptides at concentrations between 10^−4^ to 10^−9^ M to study their antiproliferation ability against cancer cells. Also, for the safety evaluation, a human skin keratinocyte cell line (HaCaT) was selected to investigate the cytotoxicity of peptides against normal human cells. All cell lines in this assay were bought from the American Type Culture Collection (ATCC, Manassas, VA, USA). 

At first, after the stock cells were revived, they were transferred into a 75 mL flask with 10 mL of the corresponding culture medium and cultured for 3–5 days at 37 °C until most cells had attached to the flask wall. Then, the culture medium was removed, and 10 mL of PBS was used to wash the wall twice and discarded. Subsequently, 3–4 mL of a trypsin solution was added for 2–3 min to detach the cells from the container wall and then 8–10 mL of FBS was added to stop the digestion reaction. The cell suspension was transferred into a 15 mL tube and centrifuged. The supernatant was removed and 4 mL of complete growth medium was added to this tube to prepare a stock cell suspension. Approximately 10 µL of a mixture containing an equal volume of trypan blue and cell medium was dropped into a chamber to measure the density of cells under the microscope. Based on the cell density, the cell suspension was diluted with foetal bovine serum (FBS) (Sigma-Aldrich, St. Louis, MO, USA) to the standard cell density (H838, MCF-7: 8 × 10^4^; U251MG: 5 × 10^4^; HCT116: 2 × 10^5^; HaCaT: 2 ×10^5^ cells/mL). Then, it was seeded into a 96-well plate at 100 μL/well. After 20–24 h incubation, 100 μL of serum-free medium was used to replace the previous FBS medium and the plate was incubated another 4 h for cell starvation. At the same time, the peptide was dissolved in DMSO at a concentration of 10^−2^ M and then mixed with the serum-free medium at working concentrations ranging from 10^−7^ to 10^−4^ M. Finally, the medium in the 96-well plate was replaced with the peptide solutions. The experimental group used 100 μL of the working concentration peptide solution. The vehicle group consisted of 1 µL of DMSO and 99 µL of serum-free medium. The blank and growth groups both comprised 100 µL of serum-free medium. The loaded 96-well plate was incubated for another 24 h for the treatment. The next day, 10 µL of MTT was added to each well and incubated for 2 h, after which, all media in each well were discarded and substituted with 100 μL of DMSO. After 10 min of shaking, the cell viability in each well was read at a wavelength of 570 nm.

### 2.12. Apoptosis Detection Assays

Fluorescent conjugates of annexin V and propidium iodide (PI) can be used to label cells undergoing cell death through the pathway of apoptosis or necrosis depending on differences in plasma membrane integrity and permeability. At the earliest stage of cell apoptosis, membrane phosphatidylserine (PS) turns from the inside of the lipid membrane to the outside. This change occurs earlier than increased cell membrane permeability. Annexin V is a phospholipid-binding protein that has a high affinity for phosphatidylserine, so it can bind to the cell membrane of early-stage apoptotic cells through the exposed phosphatidylserine on the outside of the cell. Therefore, annexin V is used as one of the sensitive indicators for detecting the early apoptosis of cells. PI does not stain live or early apoptotic cells due to the presence of an intact plasma membrane. In late apoptotic and necrotic cells, the integrity of the plasma and nuclear membranes decreases, allowing PI to pass through the membranes, intercalate into nucleic acids, and display red fluorescence. Thus, by combining annexin V with PI, cells in different stages of apoptosis can be distinguished. Therefore, when annexin V is used in combination with PI, PI is excluded from viable cells (annexin V−/PI−) and early apoptotic cells (annexin V+/PI−), while late apoptotic cells and necrotic cells show double-positive staining by FITC and PI (annexin V+/PI+).

Lung cancer line H838 cells were the tested cells in this assay and apoptosis was detected by flow cytometry. The cell apoptosis assessment utilised the Muse^TM^ Annexin V & Dead Cell Reagent (EMD Millipore, Billerica, MA, USA) following the user’s instructions. 

The H838 lung cancer cell line was cultured in a 15 mL flask as mentioned in the antiproliferation assay. When up to 80% of the cancer cells had attached to the flask wall, the complete growth medium was removed. The cells underwent two PBS washes and the PBS was discarded, after which, 3 mL of an EBSS/trypsin solution was added to detach the cells from the wall of the flask for 3 min before FBS was added to stop the digestion. This was followed by centrifugation at 300× *g* for 7 min at 4 °C, and the medium was removed. The cells were re-suspended with complete growth medium and were seeded into a 24-well plate at around 1 mL/well and 1 × 10^6^ cells/mL, and then incubated at 37 °C under 5% CO_2_ for 24 h. After the incubation, the medium was replaced with a serum-free medium for a 12 h starvation. Subsequently, the medium was discarded, 1 mL of the peptide solutions was added at concentrations of IC_10_, IC_50_, and IC_90_, and the plate was incubated for 6 h. Cells treated with 200 μM cisplatin were the positive control group. After treatment, the medium was removed and an EBSS/trypsin solution was added to detach the cells from the wall of the 24-well plate; then, the cell suspensions were transferred to 1.5 mL tubes and centrifuged again at 300× *g* for 7 min. After the trypsin solution was removed, the cells were re-suspended with PBS to a concentration of 1 × 10^6^ cells/mL. Finally, 100 μL of the cell suspension was stained with 100 μL of the Muse Annexin V & Dead Cell Reagent for at least 20 min at room temperature under dark conditions. The analysis was conducted using a Muse Cell Analyzer (EMD Millipore, Billerica, MA, USA).

The values of IC_10_ and IC_90_ were calculated according to the IC_50_ by using an online web server (https://www.graphpad.com/quickcalcs/Ecanything1/ (accessed on 1 December 2023)). The flow cytometer was used according to the users’ guide.

### 2.13. Statistical Analysis

Statistical analyses of the biological activity determination assays were conducted by using the Prism 9 software (GraphPad Software, La Jolla, CA, USA). One-way/two-way ANOVA was used to analyse the statistical significance of the difference. The data points are the mean of the independent experiments, and the error bar represents the standard error of the mean (SEM). ‘ns’ means no significant difference, * means *p* ≤ 0.05, ** means *p* ≤ 0.01, *** means *p* ≤ 0.001, and **** means *p* ≤ 0.0001.

## 3. Results

### 3.1. Structural Modifications and Physicochemical Property Analysis

The mature peptide OSTI−1949 was identified previously as an antimicrobial protein precursor [23], and the parent peptide was shown to possess modest trypsin inhibitory activity via the fully conserved hexapeptide region ‘-RCKAAFC-’ [24]. Four analogues (Table 2) were subsequently designed based on characteristic antimicrobial peptides [18]. OSTI−1716 was built by forming an artificial α-helical structure through the removal and substitution of amino acids at the flexible positions outside of the highly conserved loop, and the β-strand structure was added into OSTI−1696. As the distinctive cyclic structure of proline’s side chain was considered to be a secondary structure disruptor of alpha helices and beta sheets, the analogue OSTI−2363 was designed to form a β-strand by adjusting the first five amino acids of OSTI−1949. OSTI−2461 was generated by simply repeating the existing motif of ‘FKVH’ residues in the original sequence of the parent peptide to enhance the helicity of the secondary structure.

**Figure 1:**
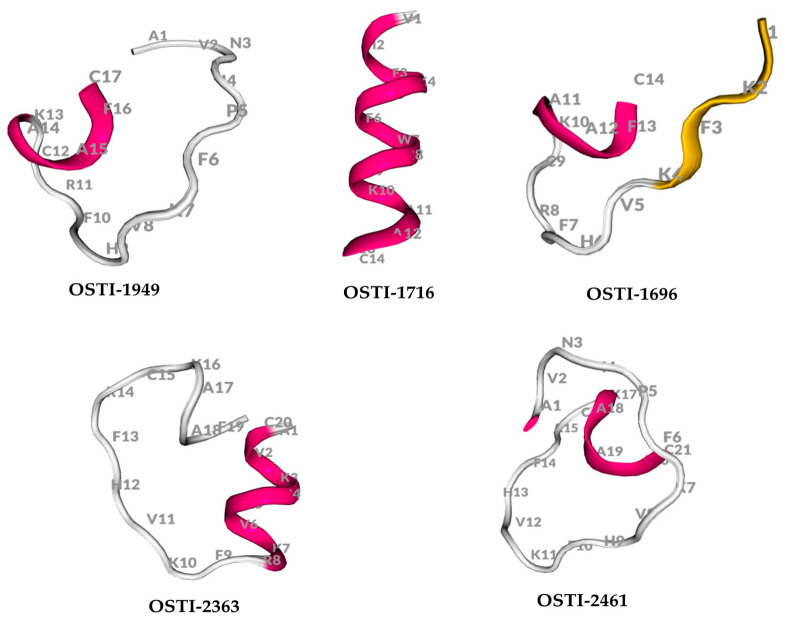
The secondary structure of peptides predicted by PEP-FOLD3.5.

The physicochemical properties of OSTI−1949 and its analogues were analysed using the BACHEM program (Table 2). Their predicted secondary structures were generated using the online server PEP-FOLD3.5 (Figure 1).

### 3.2. Peptide Synthesis and Identification

After OSTI−1949 and its four analogues were synthesised using a solid-phase peptide synthesiser, five peptides were purified by reverse-phase HPLC. The relevant chromatographs are shown in Supplementary Figure S1. Subsequently, the successful syntheses were confirmed via MALDI-TOF mass spectrometry through the analysis of their molecular mass. The mass of the analogues was identical to the theoretical mass, indicating that the peptides of interest were successfully acquired (Supplementary Figure S2).

### 3.3. Circular Dichroism (CD) Spectroscopy

**Figure 2:**
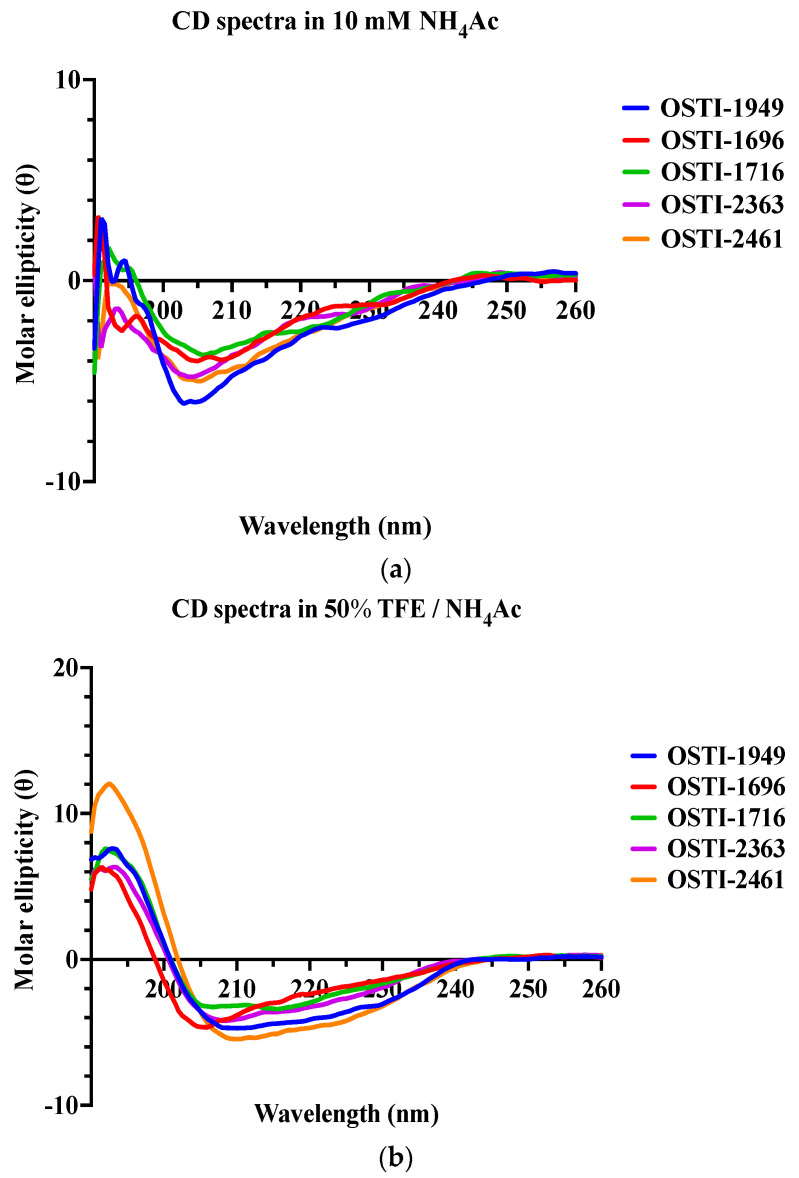
The CD spectra of OSTI−1949 and its analogues were detected in two different environments: (**a**) a 10 mM NH_4_Ac buffer that acts as an aqueous environment and (**b**) a 50% TFE/NH_4_Ac solution which mimics a microbial membrane environment.

The precise secondary structures of OSTI−1949 and its analogues were detected by circular dichroism (CD) spectroscopy in an aqueous environment, using a solution containing 10 mM ammonium acetate (NH_4_Ac), and a membrane-mimetic solution composed of 50% (*v*/*v*) trifluoroethanol (TFE) in 10 mM NH_4_Ac (Figure 2). Generally, all peptides displayed a mixed conformation of α-helices, β-strands, and random coils in both environments but tended to form more helical structures in the microbial membrane-mimetic environment. This was confirmed by the circular dichroism (CD) study, which found characteristic positive peaks at 192 nm and negative peaks at 208 nm and 222 nm in the membrane-mimetic solution (50% TFE /NH_4_Ac).

### 3.4. Trypsin Inhibition Determination

**Figure 3:**
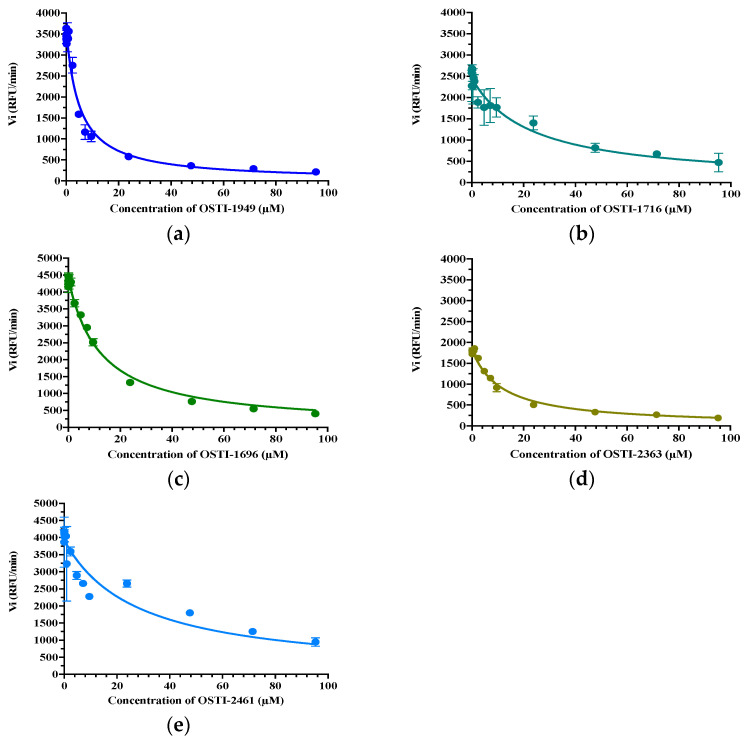
Morrison Ki plot of peptides (**a**) OSTI−1949, (**b**) OSTI−1716, (**c**) OSTI−1696, (**d**) OSTI−2363, and (**e**) OSTI−2461 against trypsin. Data points were collected from the curve through a non-linear regression analysis using GraphPad Prism 9.

The trypsin inhibitory activity of OSTI−1949 and its analogues were measured through substrate hydrolysis progress curves (Table 3). OSTI−1949 showed a moderate anti-trypsin activity with a Ki value of 2.414 μM, and this property was retained in all analogues and did not show a dramatic difference compared to the parent peptide of OSTI−1949. The Morrison inhibition plots of the tested peptides are shown in Figure 3. 

### 3.5. Antimicrobial Activity Determination

The parent peptide showed moderate antimicrobial activity against Gram-negative bacteria *E. coli* including two drug-resistant strains, *E. coli* (BAA 2340) and *E. coli* (NCTC 13846). Among the analogues, OSTI−1696 and OSTI−2363 showed the greatest improvement in antimicrobial activity against Gram-negative *E. coli* and *P. aeruginosa* and Gram-positive MRSA bacteria, especially for the drug-resistant bacterial strains of *E. coli* (Table 4).

### 3.6. Time-Killing Assays 

**Figure 4:**
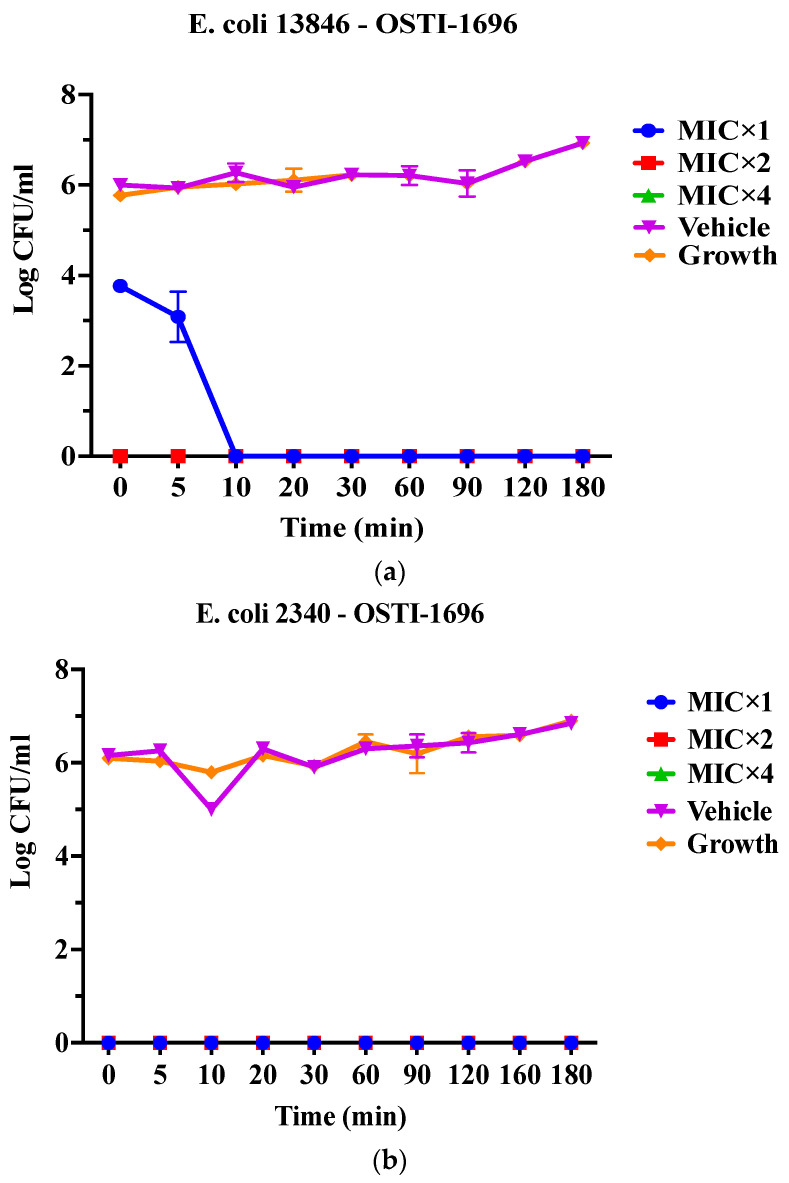

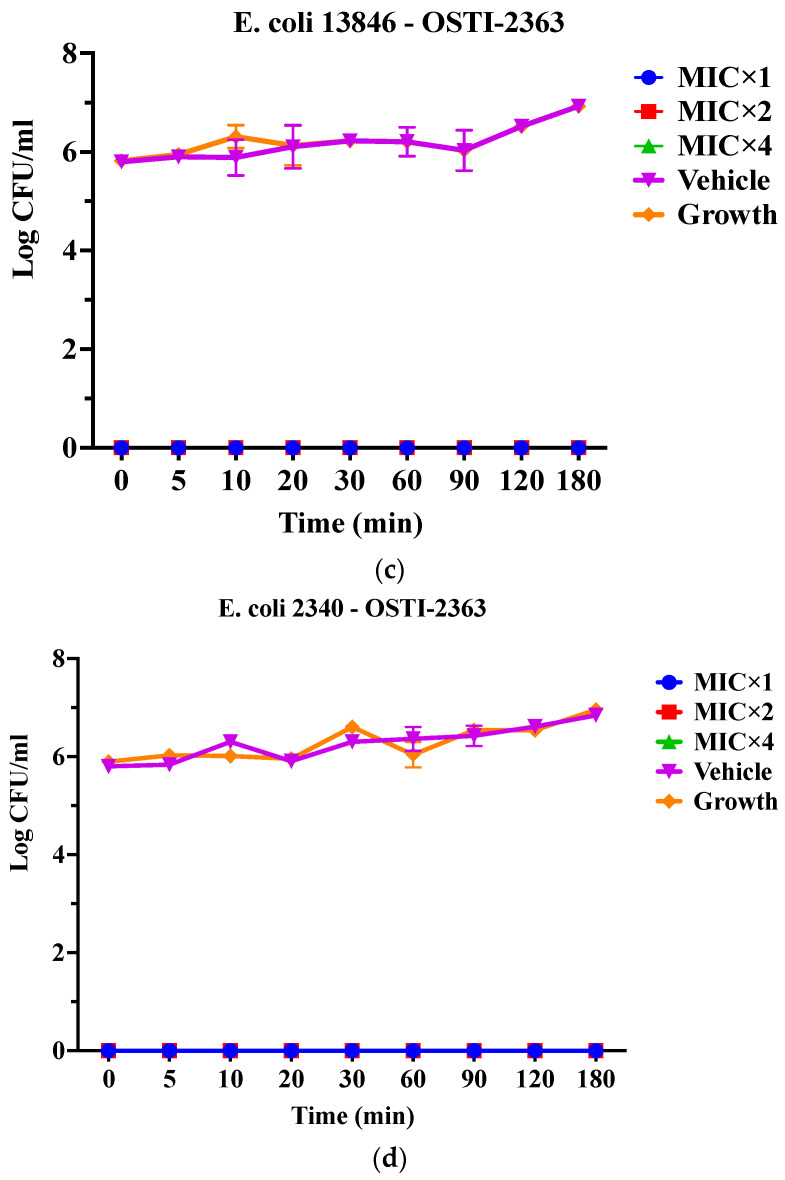
Killing kinetics of OSTI−1696 at concentrations of MIC, 2 × MIC, and 4 × MIC against *E. coli* (NCTC 13846) (**a**) and *E. coli* (BAA 2340) (**b**), and OSTI−2363 at concentrations of MIC, 2 × MIC, and 4 × MIC against *E. coli* (NCTC 13846) (**c**) and *E. coli* (BAA 2340) (**d**). Untreated bacteria were used as the positive control. The bacteria treated with broth only were used as vehicle controls. The data are shown as the mean ± standard error of the mean (SEM). **Note:** The green line is overlapped by the red line and the blue line.

As OSTI−1696 and OSTI−2363 had better antimicrobial activity, this assay aimed to investigate the kinetics of the bacteria-killing ability of the peptides at different concentrations. Generally, the two analogues displayed a very rapid killing ability against two drug-resistant bacteria at all concentrations. Apart from OSTI−1696 against *E. coli* (BAA 2340), analogues OSTI−1696 and OSTI−2363 showed almost instant bactericidal activity against the tested bacteria. OSTI−1696 could kill *E. coli* (BAA 2340) bacteria within 10 min at the MIC, and within 5 min at the concentrations of 2 × MIC and 4 × MIC (Figure 4).

### 3.7. SYTOX Green Permeability Assays

**Figure 5:**
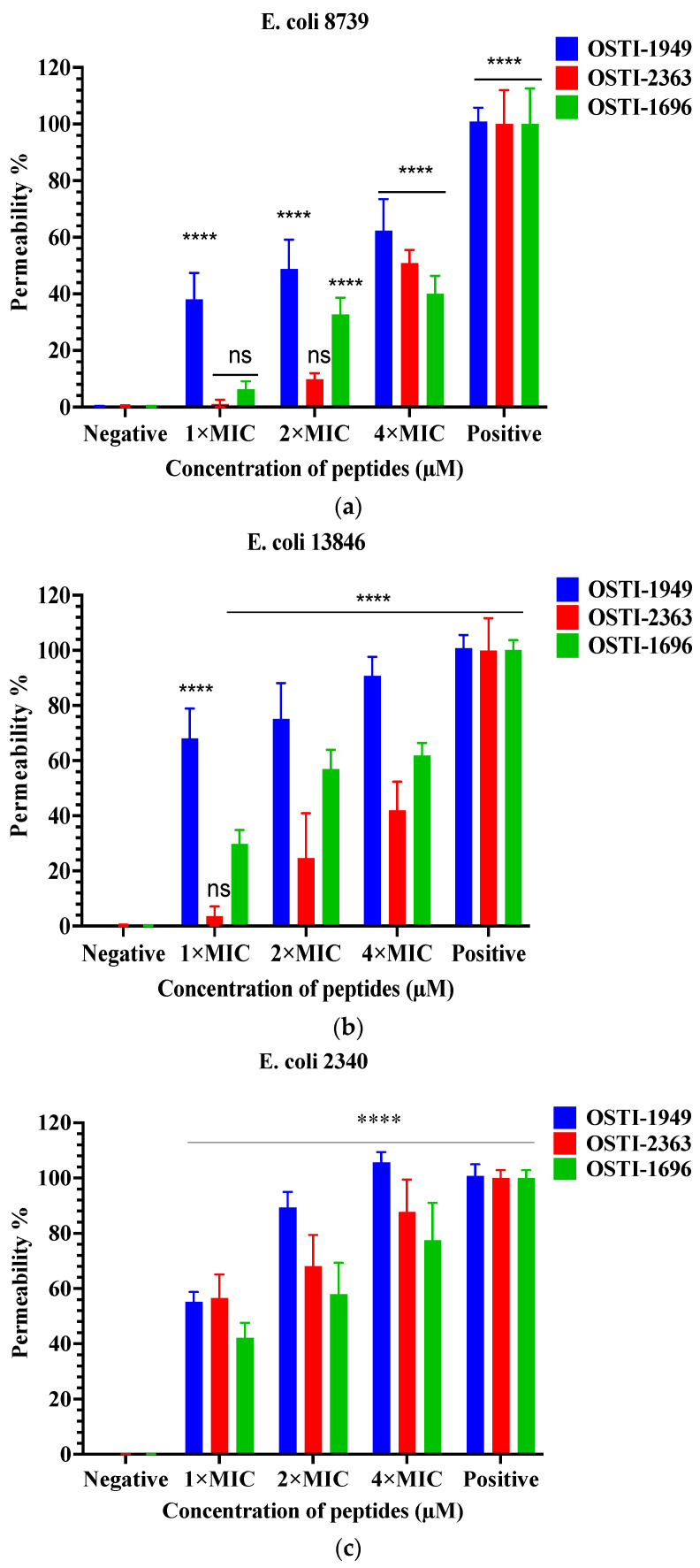
Cell membrane permeabilisation effects of peptides at the concentrations of 1 × MIC, 2 × MIC, and 4 × MIC including OSTI−1949, analogues OSTI−1696 and OSTI−2363 against *E. coli* (ATCC CRM 8739) (**a**), *E. coli* (NCTC 13846) (**b**), and *E. coli* (BAA 2340) (**c**). Bacteria incubated in TSB were used as the negative control. Bacteria treated with melittin (8 μM) were used as the positive control. The data are shown as the mean ± standard error of mean (SEM). ns means no significant difference and **** means *p* ≤ 0.0001.

The SYTOX^TM^ green assays aimed to investigate the membrane permeability of the peptides against *E. coli* strains at concentrations of MIC, 2 × MIC, and 4 × MIC (Figure 5). The parent peptide OSTI−1949 induced an approximately 50% uptake of SYTOX Green into three tested *E. coli* trains at the MIC, whereas the tested analogues displayed a large decrease in rate of membrane permeabilisation in *E. coli* (ATCC CRM 8739) and *E. coli* (NCTC 13846), and a slight decline in *E. coli* (BAA 2340).

### 3.8. Determination of In Vivo Antibacterial Activity of Analogues 

**Figure 6:**
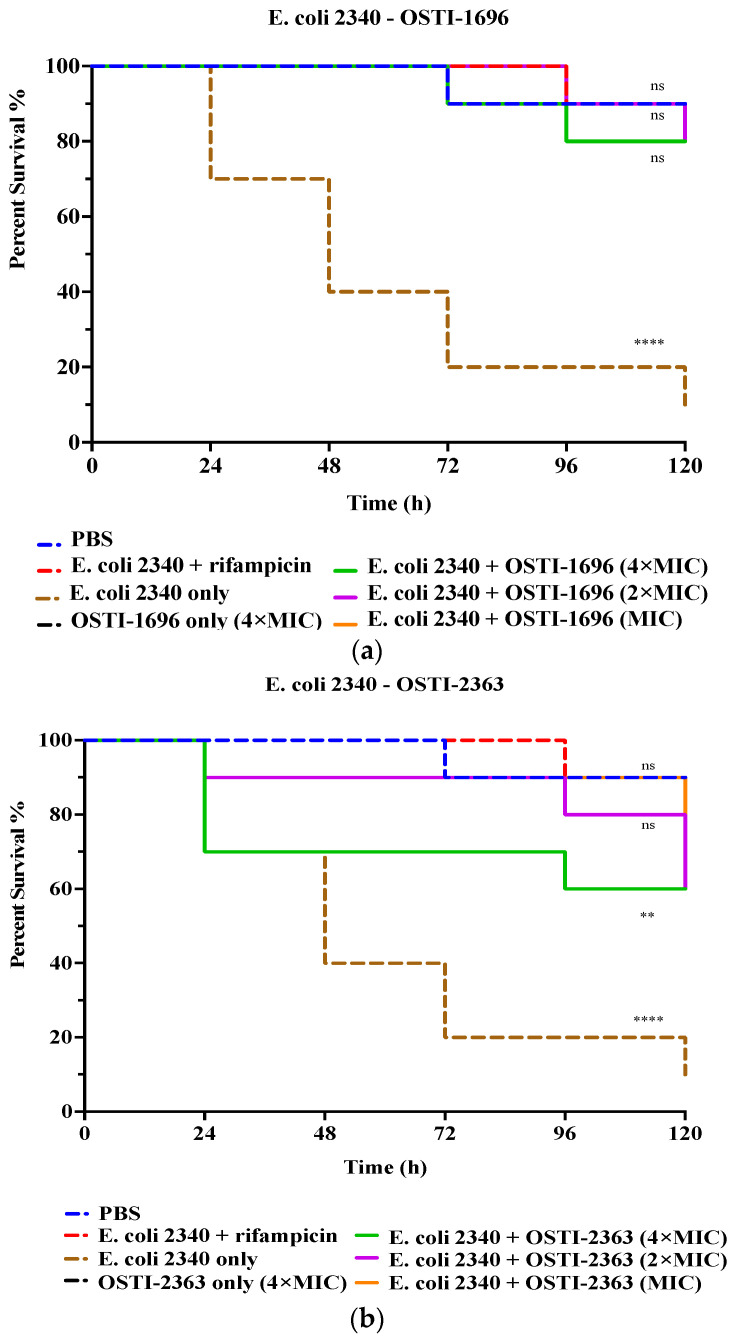
The survival curve of *E. coli* (BAA 2340)-infected waxworms treated with OSTI−1696 (**a**) and (**b**) OSTI−2363. The growth control was larvae injected with *E. coli* (BAA 2340) only. The infected larvae treated with rifampicin (20 mg/kg) were used as the positive control. Uninfected larvae treated with sterile PBS were the vehicle control, and those treated with peptides only were the cytotoxicity control. The data were analysed by one-way ANOVA, comparing the survival rates of the peptide treatment groups at different concentrations with the positive control. The significance is indicated by ns (non-significance difference), ** (*p* ≤ 0.01), and **** (*p* ≤ 0.0001).

In this study, the analogues with the best antibacterial activity in vitro were studied further for their therapeutic effect in vivo (Figure 6). The drug-resistant bacterial strain *E. coli* (BAA 2340) was used to infect waxworms as an infection model. Larvae treated with rifampicin (20 mg/kg) for 5 days were used as the positive control and exhibited a reduction in mortality to 20%. The infected larvae treated with 4 × MIC of OSTI−1696 were found to show reduced mortality (20%) after 120 h of infection, whilst the group treated with the same concentration of OSTI−2363 demonstrated 40% mortality. The survival rate did not exhibit a significant correlation with the increase in peptide concentration. It is worth noting that the survival rates of waxworms treated with the two tested peptides for 5 days achieved 80% survival, which is equivalent to the survival rate of the PBS group. 

### 3.9. Haemolysis Activities of OSTI−1949 and Its Analogues

**Figure 7:**
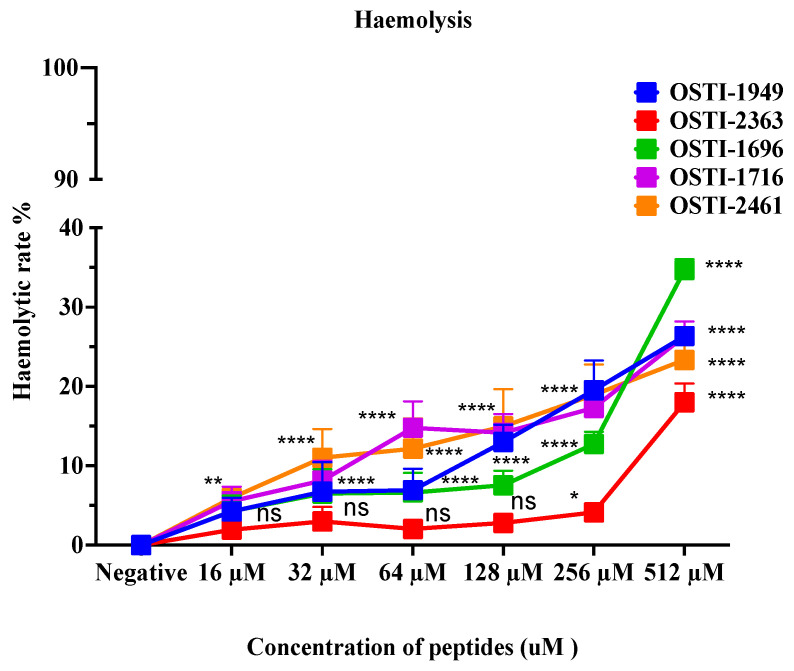
Haemolytic activity of peptides on horse erythrocytes. Data are shown as the mean ± standard error of mean (SEM)of nine replicates. The statistical significance was analysed using two-way ANOVA with Dunnett’s multiple comparisons test (comparing with the negative control): ns (non-significance difference), * means *p* ≤ 0.05, ** means *p* ≤ 0.01, and **** (*p* ≤ 0.0001).

This assay aimed to analyse the haemolytic activity on horse erythrocytes of these peptides up to a concentration of 512 μM (Figure 7). Generally, no obvious changes were observed in haemolytic activity of the analogues compared with the parent peptide. The haemolytic activity of the parent peptide OSTI−1949 was less than 10% at the MIC, and lower than 20% at a concentration of 256 μM. When the concentration was higher than 256 μM, the haemolytic activity of the analogues increased greatly, particularly OSTI−1696. The values of HC_20_ are listed in Table 5.

### 3.10. Antiproliferation Activities of OSTI−1949 and Its Analogues

**Figure 8:**
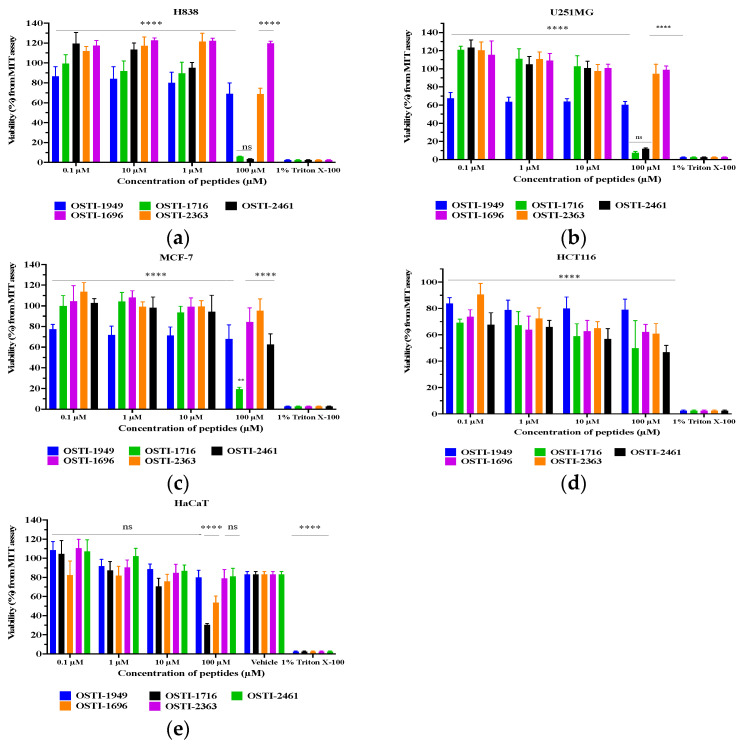
Cell viability of H838 (**a**), U251MG (**b**), MCF-7 (**c**), HCT116 (**d**), and HaCaT cells (**e**) after treatment with peptides for 24 h. The cell viability of the growth control was regarded as 100%. Data are represented as the mean and standard error of the mean (SEM) of three independent experiments. Not significant (ns), *p* ≤ 0.01 (**) and **** (*p* ≤ 0.0001). Compared to the group treated with 1% Triton X-100, which were determined by two-way ANOVA.

MTT assays were performed to investigate the antiproliferation activities of the parent peptide and its analogues (Figure 8). The IC_50_ values of the peptides against the tested cell lines are shown in Table 6. The parent peptide OSTI−1949 did not exhibit any antiproliferation ability against either cancer cells or normal cells, whilst the modified peptides OSTI−1716 and OSTI−2461 displayed significant improvements in antiproliferative activity against the tested cancer cell lines. Notably, a significant improvement was observed for OSTI−1716 against the normal cell line HaCaT at the highest tested concentration of 100 μM. Similarly, OSTI−1696 and OSTI−2363 also displayed notable improvements against HaCaT at 100 μM. Additionally, OSTI−2461 not only exhibited potent anticancer activity against cancer cell lines, but it also had minimal impact upon the normal cell line HaCaT. 

### 3.11. Apoptosis Assays

**Figure 9:**
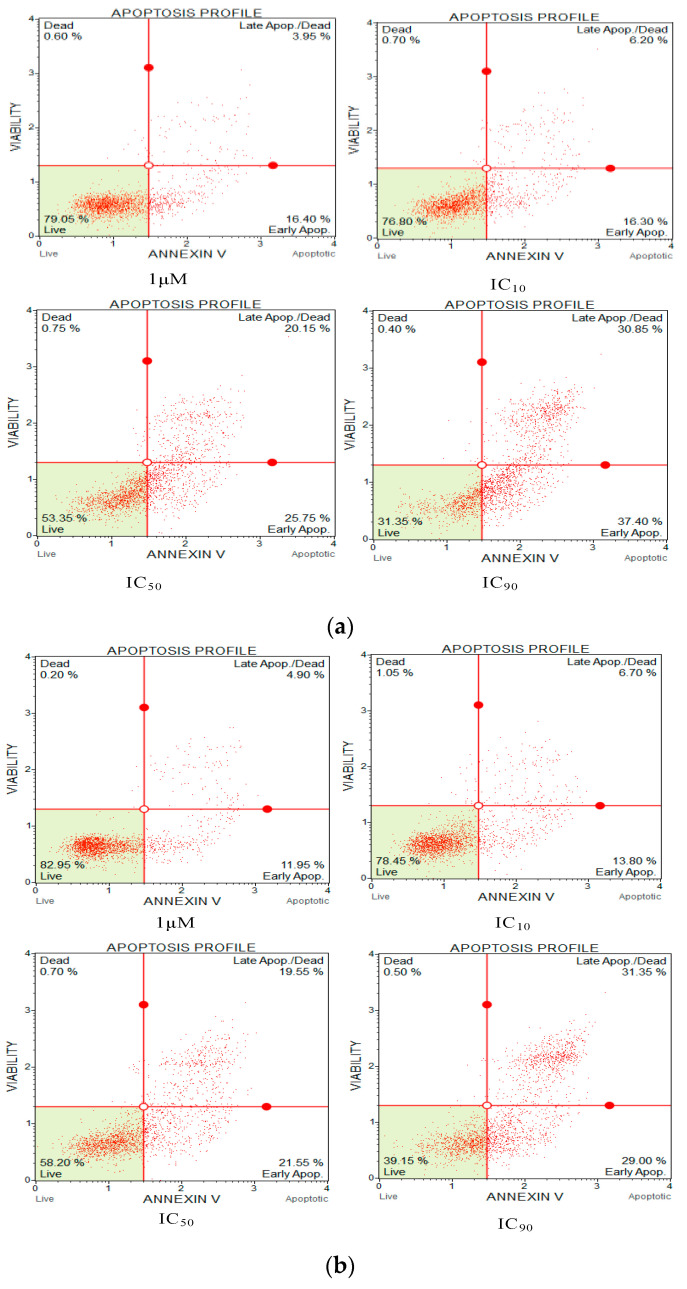

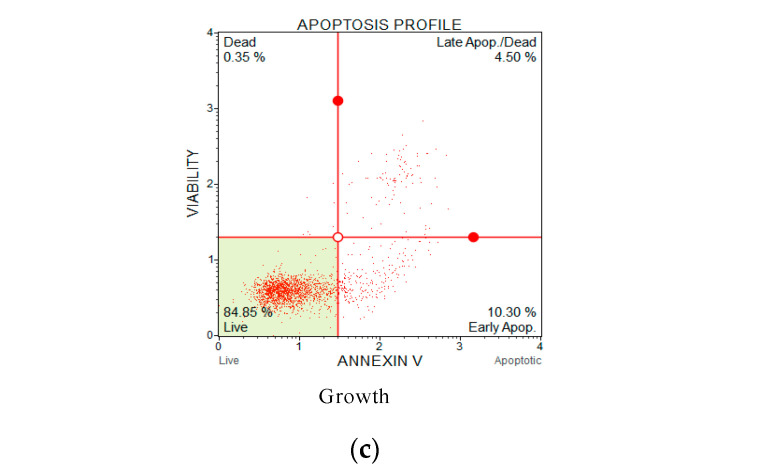
Flow cytometric analysis of annexin V/PI staining. The H838 prostate cancer cells were treated with peptides OSTI−1716 (**a**) and OSTI−2461 (**b**) for 6 h; the cells treated with serum-free medium acted as the growth control (**c**). Cells were dyed with annexin V/PI and analysed by flow cytometry. The four-quadrant diagram shows dead cells (annexin V−/PI+), live cells (annexin V−/PI−), cells in post-apoptotic necrosis or late apoptosis (annexin V+/PI+), and cells in early apoptosis (annexin V+/PI−).

Annexin V and propidium iodide (PI) apoptosis assays were used to study the ratio of cellular apoptosis in the cancer cell line H838 following treatment with OSTI−1716 and OSTI−2461 for 6 h since these analogues showed the greatest antiproliferative activity towards lung cancer cell line H838 among the tested cancer cell lines (Figure 9). After accounting for the interference from the growth control cells, the percentage of H838 cells in the annexin V+/PI− category (cells in early apoptosis) increased significantly in three analogue treatments. The percentage of apoptotic cells in the OSTI−1716 and OSTI−2461 treatment groups increased from 16.40% to 37.40% and from 11.95% to 29.00%, respectively. In contrast, the growth control exhibited a lower percentage of apoptotic cells at 10.30%. Both analogues induced early apoptosis at the tested concentration, and in a dose-dependent manner. The cancer cells treated with peptides at IC_90_ displayed the highest early apoptosis ratio. 

## 4. Discussion

Trypsin inhibitors (TIs) possess the capability of binding competitively to trypsin in a substrate-like action. This binding mechanism allows TIs to effectively regulate the activity of trypsin [25]. These inhibitors enjoy a broad distribution and are prevalent in both plants [26] and animals [27,28], and their significance extends into clinical applications, where they can be used in cancer prevention [29], obesity treatments [30], and therapy for coeliac disease [31].

Amphibian skin secretions are known to be an abundant source of multifunctional bioactive peptides. These peptides serve as valuable agents, contributing to hydration and playing pivotal roles in regulating and fortifying defensive mechanisms [32]. In recent decades, numerous protease inhibitors with low molecular weights and high inhibition efficiency have been discovered in amphibian secretions. These inhibitors are considered promising candidates for the development of novel peptide drugs [25]. 

Based on shared characteristics such as structural domains, sequence homology, reactive site similarities, and mechanistic properties, these inhibitors can be categorised into distinct types, including but not limited to Bowman–Birk- [33], Kunitz- [34], and Kazal-type inhibitors [35]. For instance, Bowman–Birk inhibitors were identified in Pelophylax frogs [36], Kunitz-type inhibitors have been isolated from both tomato [27] and ranid frogs [8], and Kazal-type inhibitors were extracted from Phyllomdusa frogs [37]. To date, the Kunitz-type inhibitors have earned the least attention among these three inhibitor types.

Within the extensive array of identified Kunitz-type inhibitors, those derived from animals hold particular importance in the exploration of structure–activity relationships [36,38,39,40]. Generally, this kind of inhibitor possesses a fully conserved hexapeptide loop region ‘-RCKAAFC-’ and modest trypsin inhibitory activity. All synthetic peptides exhibited a comparable level of anti-trypsin activity when the trypsin inhibitory loop of “-RCKAAFC-” was maintained in the amino acid sequence of the analogues. The minimal variations in the Ki values among the different analogues suggest that residues outside of the trypsin inhibitory loop might also influence the trypsin inhibition activity, although to a lesser extent [41,42]. 

In this study, a mature peptide of the Kunitz type, OSTI−1949 from *Odorrana schmackeri*, was assessed. It showed a moderate trypsin inhibitory activity, with a Ki value of 2.414 μM, which is consistent with the value reported in previous research [10,24]. OSTI−1949 also exhibited a moderate, but broad-spectrum, antimicrobial activity against Gram-negative bacteria such as *E. coli*, *P. aeruginosa*, and *K. pneumoniae*. Additionally, it also displayed slight antimicrobial activity against the Gram-positive bacteria MRSA. However, no apparent antiproliferative activity against the selected cancer cell lines was observed. Several analogues were purposefully designed with the aim of enhancing the biological activities of OSTI−1949, such as the antimicrobial and/or anticancer activity, while maintaining its trypsin inhibition activity. The strategies used to structurally alter OSTI−1949 drew their inspiration from the characteristics of other antimicrobial peptides [18]. 

Regarding antimicrobial activity, the majority of the analogues demonstrated an enhancement in their antimicrobial effects. Notably, OSTI−2363 and OSTI−1696 exhibited the most substantial improvements in antimicrobial activity in vitro against Gram-negative bacteria strains and the Gram-positive bacteria strain MRSA. Moreover, they exhibited the highest antibacterial efficacy against the drug-resistant bacteria strain *E. coli* (BAA 2340) in infected waxworms, with no significant difference compared to rifampicin. It is well-known that the secondary structure represents one of the crucial parameters in the evaluation of antimicrobial peptides (AMPs). The secondary structure serves as the fundamental element in the interaction between antimicrobial peptides (AMPs) and the membrane. The process of folding the peptide into a regular secondary structure is pivotal as it facilitates the insertion of the AMP into the bacterial membrane [43,44]. Typically, many AMPs exhibit a disordered structure in aqueous solutions. When introduced into a membrane environment, the peptides can interact with phospholipid biomolecules, resulting in the formation of a highly stabilised secondary structure, and this transition is of immense significance for the antimicrobial activity of these peptides [45]. In addition, the distinct amphipathic nature inherent in the α-helix and ß-sheet structures contributes significantly to the antimicrobial efficacy of AMPs. This characteristic enables an electrostatic interaction between the peptide’s hydrophilic surface and the negatively charged elements of bacterial membranes. Simultaneously, the peptide’s hydrophobic surface integrates into the membrane, increasing its permeability [46,47]. In this research, the creation of an artificial secondary structure, comprising α-helices and ß-sheets at the C-terminal of Kunitz peptides, enhanced the interaction of the trypsin inhibition loop with bacterial membranes. This modification could underlie the observed enhancement in the antimicrobial activity of the analogues. Additionally, the analogues OSTI−2363 and OSTI−1696 demonstrated remarkable efficacy in killing bacteria. OSTI−2363 displayed a rapid bactericidal activity, with the selected bacterial strains being eradicated within 5 min, even at the lowest tested concentration. OSTI−1696 exhibited bactericidal ability within 10 min at the MIC, demonstrating instant bacterial-killing potency at 2 × MIC and 4 × MIC concentrations. The antibacterial characteristics of these analogues differ from those of classical antimicrobial peptides (AMPs), which possess a bactericidal ability with slow kinetics [48,49,50,51]. The enhanced antibacterial kinetics observed in the modified peptides suggest a potential contribution of trypsin inhibitory activity to this property. 

The subsequent investigation into membrane permeability induced by these peptides using the SYTOX green permeability assays revealed intriguing findings. Notably, OSTI−2363 and OSTI−1696 exhibited a reduction in permeability rates to some extent compared to the parent peptide particularly at the MIC, except for OSTI−2363 against *E. coli* (BAA 2340).

Protease inhibitors have held a prominent role in anticancer research [52,53]. To date, there has been no exploration into the anticancer potential of the Kunitz-type inhibitor OSTI−1949, and therefore, its anticancer activity has not been studied. The original peptide OSTI−1949 did not exhibit obvious antiproliferative activity against both cancer and normal cells, whereas the analogues, particularly OSTI−1716 and OSTI−2461, demonstrated significant enhancements in antiproliferative activity against a variety of cancer cells. Commonly, anticancer peptides (ACPs) are characterised by their small size containing fewer than 50 amino acids. They usually exhibit a cationic nature [54], and their antiproliferative effects are typically performed via attaching to negatively charged phosphatidylserines that are uncovered on the surfaces of cancer cells [51,52]. Indeed, a significant number of ACPs are derived from antimicrobial peptides (AMPs). As a result, both AMPs and ACPs share similar characteristics, including cationic properties, often containing basic and hydrophobic residues. This similarity underscores the potential dual functionality of these peptides in both antimicrobial and anticancer activities [54,55]. 

The negatively charged surface of cancer cells, akin to bacterial cells, renders them susceptible to the actions of many AMPs. The broad-spectrum toxicity observed against both bacterial and cancer cells is often caused by the electrostatic interaction between the cationic nature of these peptides and the negatively charged components of the plasma membrane of cancer cells. This electrostatic interaction is considered pivotal in the cancer-selective toxicity of ACPs [56]. Numerous studies have discovered that the mode of action for most ACPs parallels that of AMPs, involving a membrane-lytic mechanism [55,56,57]. While the membrane-lytic mode of action is predominant among ACPs, some have demonstrated alternative mechanisms such as the induction of apoptosis through the disruption of mitochondrial membranes. The selective toxicity of many ACPs against cancer cells is often linked to variations in lipid content and other components within the biological membranes of normal versus cancer cells [54,55].

The annexin V/PI staining assay was carried out to explore the mechanism of cell death of peptide-treated lung cancer H838 cells by flow cytometry. Compared with the growth control, treatment with the two analogues meaningfully increased the proportion of H838 cells in the annexin V+/PI− category. Early apoptotic cells in the OSTI−1716 and OSTI−2461 treatment groups increased from 16.40% to 37.40% and 11.95% to 29.00%, respectively, at the different concentrations of analogues after a 6 h incubation, with the growth control at 10.30%. The apoptosis results from the annexin V/PI assay suggested that the modified peptides could induce H838 programmed death, and it was in a dose-dependent manner.

Furthermore, the relatively low toxicity of the analogues OSTI−1696 and OSTI−2363 toward erythrocytes, along with their minimal antiproliferative effects on the HaCaT cell line, suggests that these analogues are safe for normal cells in vitro. Moreover, the bioavailability of peptides holds significant importance in clinical applications, particularly in evaluating their efficacy against bacterial infections. Employing Galleria mellonella larvae (waxworms) as an in vivo bacterial infection model provides a valuable means to assess the effectiveness of peptides in combating infections. In this study, when compared to sterile PBS, there was no significant difference in the survival rate of waxworm larvae treated with the two analogues at concentrations up to 4 × MIC. This suggests that their safety profile is promising for further evaluation in therapeutic applications. 

However, despite OSTI−1761 showing notably enhanced anticancer activities, its haemolytic activity remained stable, with a slight reduction compared to OSTI−1949. Interestingly, it exhibited inhibition against HaCaT cells, suggesting potential cytotoxic effects on normal cells despite its anticancer efficacy. In contrast, OSTI−2461 emerged as a particularly promising analogue, demonstrating potent anticancer activity alongside lower toxicity on both normal cells and erythrocytes. These features make OSTI−2461 a promising candidate for further investigation and potential therapeutic development.

## 5. Conclusions

In summary, the successful generation of multifunctional modified peptides was achieved through the creation of artificial secondary structures, incorporating both α-helix and ß-sheet formations. These peptides demonstrated robust trypsin inhibitory activity along with remarkable antimicrobial or antiproliferative efficacy against cancer cells. Moreover, they exhibited good safety profiles regarding haemolysis and the inhibition of normal cell proliferation. This suggests that structural modification of protease inhibitor peptides is an effective strategy for enhancing the properties of peptides.

## Figures and Tables

**Table 1:** The amino acid sequences of the novel peptide and its modified analogues.

No.	Peptides	Sequences
1	OSTI−1949	AVNIPFKVHFRCKAAFC
2	OSTI−1716	VIFKVFWRCKAAFC
3	OSTI−1696	IKFKVHFRCKAAFC
4	OSTI−2363	AVKRAVKRFKVHFRCKAAFC
5	OSTI−2461	AVNIPFKVHFKVHFRCKAAFC

**Table 2:** The physicochemical properties of OSTI−1949 and its analogues.

Peptide	Sequence	Hydrophilicity	Net Charge
OSTI−1949	AVNIPFKVHFRCKAAFC	−0.42	3.0
OSTI−1716	VIFKVFWRCKAAFC	−0.69	2.91
OSTI−1696	IKFKVHFRCKAAFC	−0.16	4.0
OSTI−2363	AVKRAVKRFKVHFRCKAAFC	0.23	7.0
OSTI−2461	AVNIPFKVHFKVHFRCKAAFC	−0.41	4.09

**Table 3:** Inhibitory activity of OSTI−1949 and its analogues against trypsin.

Peptide	Sequence	Trypsin Ki (μM)
OSTI−1949	AVNIPFKVHFRCKAAFC	2.414
OSTI−1716	VIFKVFWRCKAAFC	11.15
OSTI−1696	IKFKVHFRCKAAFC	6.077
OSTI−2363	AVKRAVKRFKVHFRCKAAFC	5.596
OSTI−2461	AVNIPFKVHFKVHFRCKAAFC	13.47

**Table 4:** MIC/MBC values of peptides against ten microorganisms.

MIC/MBC (μM)						
Microbial Strain		OSTI−1949	OSTI−1716	OSTI−1696	OSTI−2363	OSTI−2461
Gram-negative bacteria	*E. coli* (ATCC CRM 8739)	32/32	32/32	8/8	4/16	16/16
	*E. coli* (BAA 2340)	32/32	32/32	4/4	8/8	16/16
	*E. coli* (NCTC 13846)	32/32	32/32	4/4	8/8	8/16
	*P. aeruginosa* (ATCC CRM 9027)	64/128	64/64	8/16	16/16	16/16
	*K. pneumoniae* (ATCC CRM 43861)	64/128	128/128	64/64	32/32	16/32
	*A. baumannii* (BAA 747)	>512	512/512	256/256	256/256	>512
Gram-positive bacteria	*S. aureus* (ATCC CRM 6538)	>512	>512	512/512	>512	>512
	*E. faecium* (NCTC 12697)	>512	>512	>512	>512	>512
	MRSA (NCTC 12493)	256/256	32/32	32/32	16/16	16/32
Yeast	*C. albicans* (ATCC 10231)	>512	>512	>512	>512	>512

**Table 5:** HC_20_ of peptides against horse erythrocytes.

Peptide	HC_20_ (μM)
OSTI−1949	287.8
OSTI−1716	270.4
OSTI−1696	297.7
OSTI−2363	555.4
OSTI−2461	304.2

“HC_20_” is the peptide concentration that led to 20% haemolysis of red blood cells.

**Table 6:** IC_50_ of peptides against cancer cell lines.

IC_50_ (μM)					
Peptide	H838	U251MG	MCF-7	HCT116	HaCaT
OSTI−1949	N.I.	N.I.	N.I.	N.I.	N.I.
OSTI−1716	27.39	81.10	45.14	N.I.	28.57
OSTI−1696	N.I.	N.I.	238.4	N.I.	906.1
OSTI−2363	115.1	N.I.	N.I.	N.I.	N.I.
OSTI−2461	29.74	88.25	169.0	N.I.	N.I.

“N.I.” means no obvious inhibitory effect.

## Data Availability

The data presented in this study are available in NCBI-BLAST at https://www.ncbi.nlm.nih.gov/protein/ADE48807.1?report=genbank&log$=protalign&blast_rank=4&RID=2RS9KGUR013, reference number [23]. These data were derived from the following resources available in the public domain: PubMed (https://pubmed.ncbi.nlm.nih.gov/).

## Supplementary Materials

The following supporting information can be downloaded at: https://www.mdpi.com/article/10.3390/pharmaceutics16050597/s1, Figure S1: Purity of (a) OSTI−1949, (b) OSTI−1716, (c) OSTI−1696, (d) OSTI−2363, and (e) OSTI−2461 was confirmed by RP-HPLC; Figure S2: MALDI-TOF MS spectrometry of the five synthetic peptides: (a) OSTI−1949, (b) OSTI−1716, (c) OSTI−1696, (d) OSTI−2363, and (e) OSTI−2461.
