# Supplementary material for: An Effective Modification Strategy to Build Multifunctional Peptides Based on a Trypsin Inhibitory Peptide of the Kunitz Family
Source: Pharmaceutics. 2024 Apr 27;16(5):597. doi: 10.3390/pharmaceutics16050597 (PMC11125039; doi:10.3390/pharmaceutics16050597)
Supplement: Supplementary file 1 [file pharmaceutics-16-00597-s001.zip › pharmaceutics-2953059-supplementary.pdf]

Supplementary Materials: An Effective Modification Strategy to Build Multifunctional Peptides Based on a Trypsin Inhibitory Peptide of the Kunitz Family

Ying Wang, Daning Shi, Wanchen Zou, Yangyang Jiang, Tao Wang, Xiaoling Chen, Chengbang Ma, Wei Li, Tianbao Chen, James F. Burrows, Lei Wang and Mei Zhou

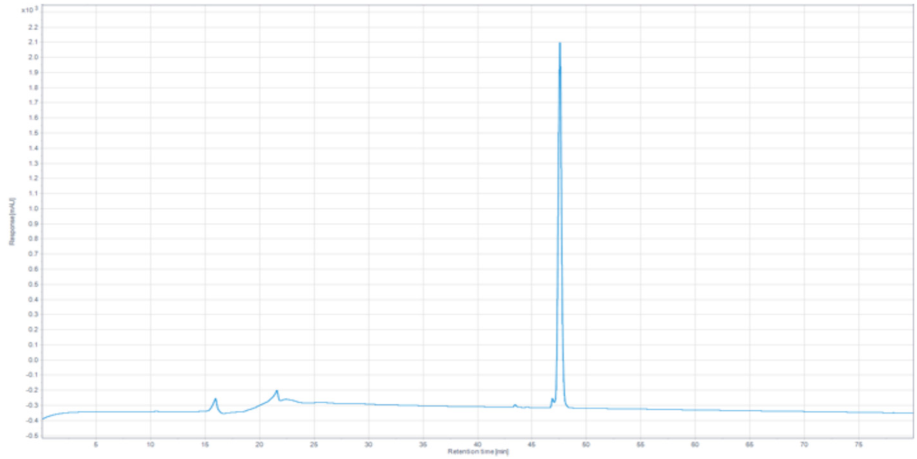

(**a**)

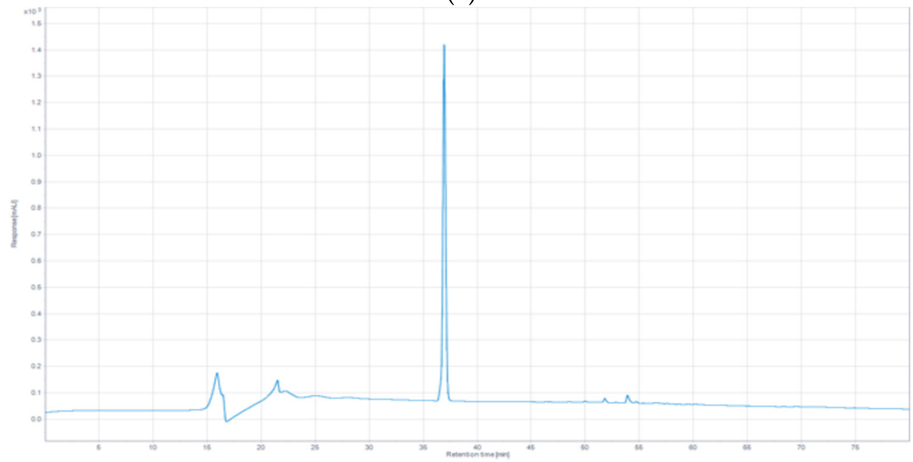

(**b**)

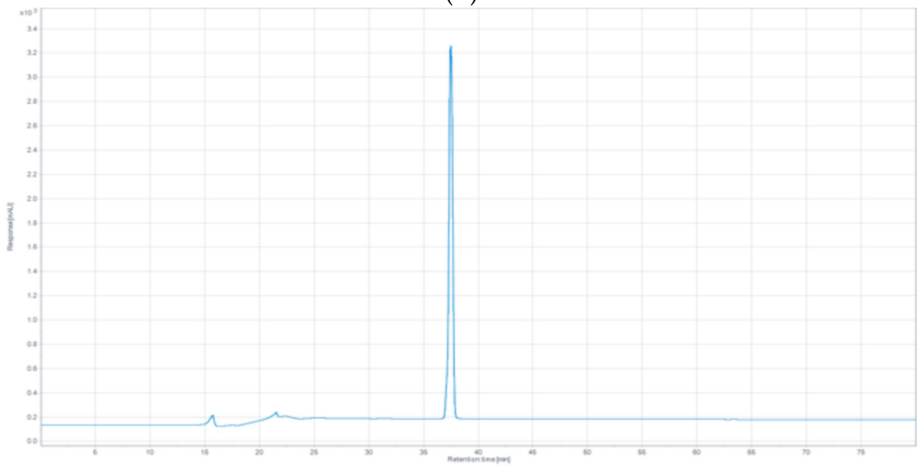

(**c**)

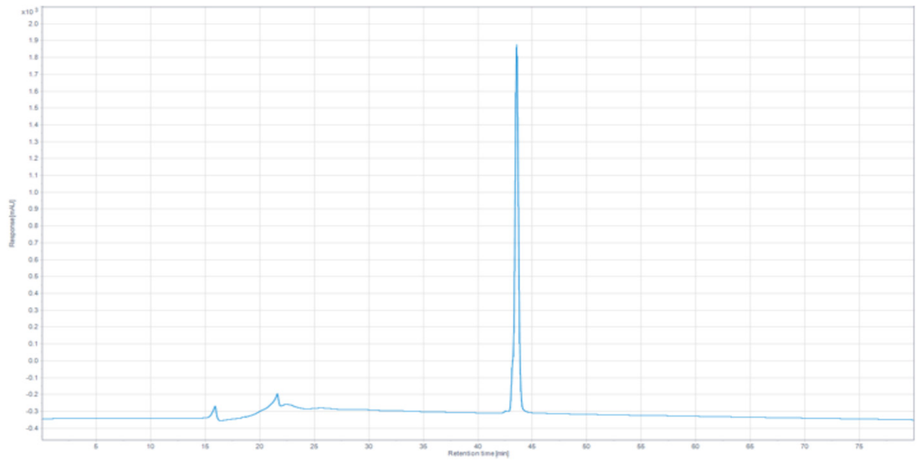

(**d**)

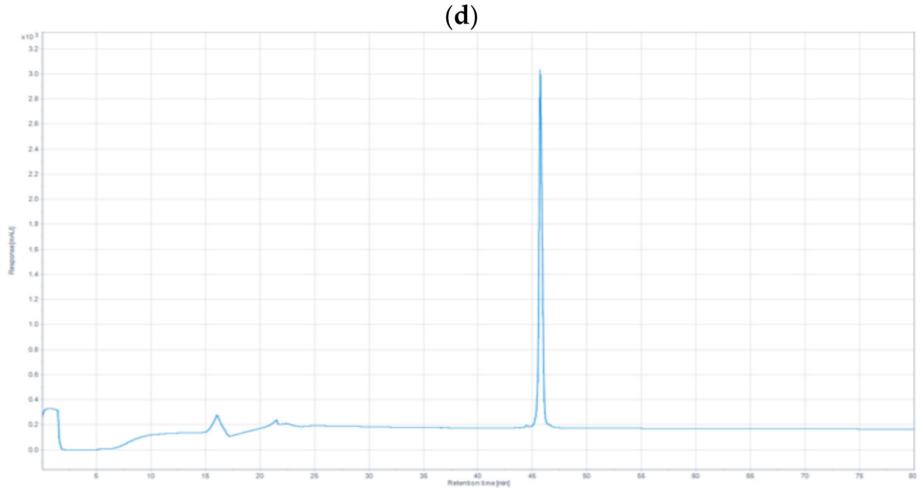

(**e**)

Figure S1: Purity of (**a**) OSTI-1949, (**b**) OSTI-1716, (**c**) OSTI-1696, (**d**) OSTI-2363, and (**e**) OSTI-2461 was confirmed by RP-HPLC.

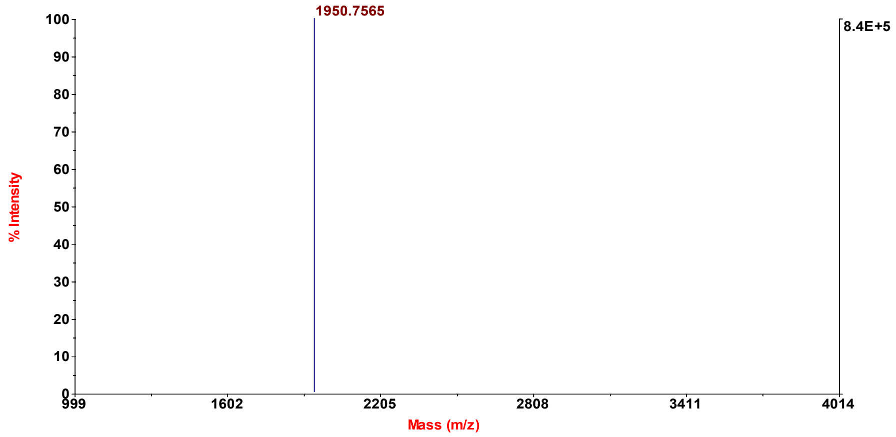

(**a**)

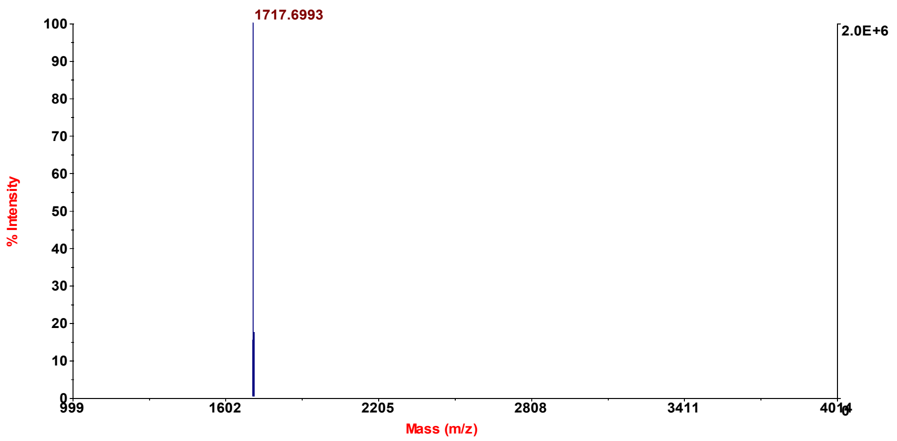

1717.6993
2.0E+6
% Intensity
100
90
80
70
60
50
40
30
20
10
0
999
1602
2205
2808
3411
4014
Mass (m/z)

(b)

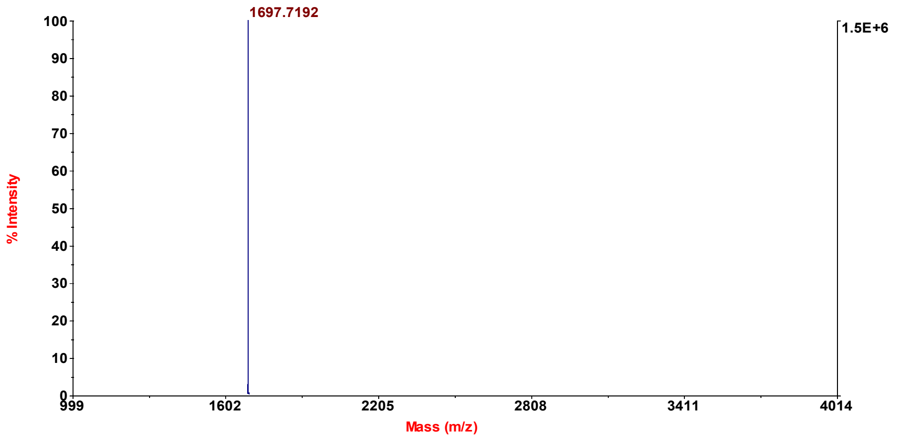

1697.7192
1.5E+6
% Intensity
100
90
80
70
60
50
40
30
20
10
0
999
1602
2205
2808
3411
4014
Mass (m/z)

(c)

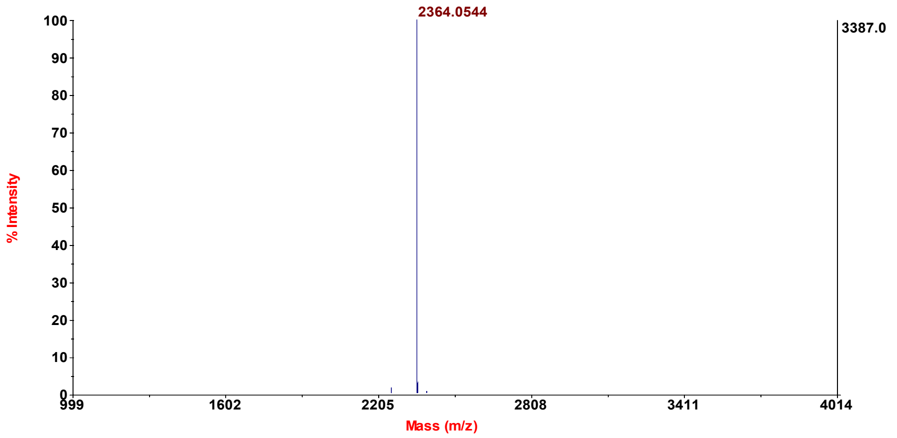

2364.0544
3387.0
% Intensity
100
90
80
70
60
50
40
30
20
10
0
999
1602
2205
2808
3411
4014
Mass (m/z)

(d)

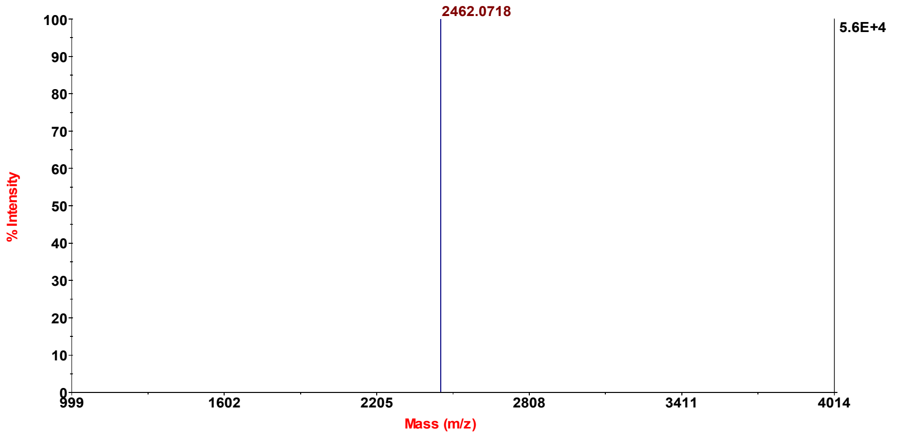

2462.0718
5.6E+4
% Intensity
100
90
80
70
60
50
40
30
20
10
0
999
1602
2205
2808
3411
4014
Mass (m/z)

(**e**)

Figure S2: MALDI-TOF MS spectrometry of the five synthetic peptides: (**a**) OSTI-1949, (**b**) OSTI-1716, (**c**) OSTI-1696, (**d**) OSTI-2363 and (**e**) OSTI-2461.
